# The Role of Classmates’ Modeling and Reinforcement in Adolescents’ Perceived Classroom Peer Context

**DOI:** 10.1007/s10964-020-01325-8

**Published:** 2020-10-03

**Authors:** Esther C. A. Mertens, Maja Deković, Monique Van Londen, Ellen Reitz

**Affiliations:** Utrecht University, Child and Adolescent Studies, Heidelberglaan 1, 3584 CS Utrecht, The Netherlands

**Keywords:** Peer influence, Perceived classroom peer context, Dyadic mutuality, Modeling, Reinforcement, Classmates

## Abstract

Experiences with classmates can affect adolescents’ academic, emotional, and social development. The aim was to examine whether changes in classmates’ modeling and reinforcement, induced by an intervention, affected changes in adolescents’ perceived classroom peer context and whether these associations were moderated by dyadic mutuality. Questionnaires and observations were used in a sample of 7th Grade students (*N* = 152; *M*_*age*_ = 12.37; 53.8% boys). Generally, changes in classmates’ modeling and reinforcement were unrelated to adolescents’ perceived classroom peer context, except for classmates’ prosocial modeling. Increases in prosocial modeling were related to decreases in victimization, especially for dyads with high levels of mutuality. The results suggest that classmates’ prosocial modeling may be more important for the perceived classroom peer context than classmates’ deviant modeling.

## Introduction

The classroom is an important developmental context for adolescents in which experiences with peers affect their academic, emotional, and social development (Rubin et al. 2006). It is therefore important that adolescents perceive the classroom peer context as positive and safe. This means that students should feel comfortable around their classmates, feel included in the group, and experience few conflicts in the classroom (Boor-Klip et al. 2016). In particular, lack of victimization by bullies is an important aspect of a positive perception of the classroom peer context, given that victimization is strongly associated with negative social experiences at school and perceiving the school context as dangerous (Goldstein et al. 2008). Not all students experience the peer context in the classroom as positive. For instance, in the Netherlands 20% of the adolescents following the preparatory vocational education track (one of the three educational tracks in the Dutch secondary school system) experience problems with their peers (Stevens and De Looze 2018). Therefore, the aim of the present study was to examine the processes through which adolescents’ experienced peer context in the classroom is influenced by their classmates. The focus was on how adolescents perceive their classmates in general and specifically on experienced victimization by the adolescents themselves.

### Modeling and Reinforcement

According to the social learning theory (Bandura 1978), two major ways through which peer influences occur are modeling and reinforcement. Through modeling adolescents learn new social skills and behavioral tendencies by observing their peers. They look at their peers’ behaviors and the positive or negative consequences these peers encounter. When certain behavior of peers has positive consequences, the adolescent imitates that behavior. When certain behavior of peers has negative consequences, it is less likely that the adolescent imitates that behavior. The second mechanism is reinforcement. Through reinforcement adolescents learn new behaviors and tendencies based on positive feedback of peers. Peers respond positively to certain behavior of the adolescent (e.g., by laughing, agreeing, giving a thumps up). This positive feedback increases the chance that the adolescent shows this behavior again.

Peers’ modeling and reinforcement can negatively and positively influence how adolescents perceive the classroom peer context. Peers can model behavior that violates community or societal rules, i.e., deviant modeling, or positively evaluate such deviant behaviors, i.e., deviant reinforcement (Piehler and Dishion 2007). Increases in both deviant modeling and reinforcement by peers have consistently been linked to increases in adolescents’ own deviant behaviors such as aggression, antisocial behavior (e.g., Dishion and Tipsord 2011), and victimization (e.g., Ando et al. 2005). These behaviors negatively affect the classroom peer context. In contrast, peers can model behavior according to prosocial values, principles, and actions with the intention to benefit others, i.e., prosocial modeling, or respond positively to such prosocial behaviors, i.e., prosocial reinforcement (Piehler and Dishion 2007; Memmott-Elison et al. 2020). Increases in prosocial modeling and reinforcement by peers have been linked to decreases in adolescents’ own problem behaviors, for instance, antisocial behaviors (e.g., Hofmann and Müller 2018), aggression, and depression (Memmott-Elison et al. 2020), but also to increases in adolescents’ own prosocial behaviors (Busching and Krahé 2020), positive interpersonal interactions in the class (e.g., Telzer et al. 2018), and prosocial goal pursuit (e.g., Barry and Wentzel 2006). This can positively affect the classroom peer context. Thus, increases in classmates’ deviant modeling and reinforcement may have a negative impact on adolescents’ perceptions of the classroom peer context, whereas increases in classmates’ prosocial modeling and reinforcement may have a positive impact.

### Dyadic Mutuality

The relation between classmates’ modeling or reinforcement and the perceived classroom peer context may be dependent on dyadic mutuality. Dyadic mutuality indicates the degree of responsiveness, reciprocity and understanding shared between individuals. In dyads with high levels of mutuality, peers listen and respond appropriately to each other, are genuinely interested in one another, and express affection towards each other (Piehler and Dishion 2007). Dyadic mutuality refers to the *process* of interaction and describes *how* individuals interact, regardless of content (Harrist and Waugh 2002). Although dyadic mutuality is related to positive (e.g., satisfaction, intimacy) and negative aspects (e.g., conflict, dissatisfaction) of friendship (Piehler and Dishion 2007), it is not the same; “dyadic mutuality” indicates the interaction-style, whereas “friendship” indicates a specific type of relationship between individuals (Harrist and Waugh 2002).

Berndt (2002) theorizes that adolescents are more strongly influenced by peers with whom they have high quality interactions, which is supported by empirical research (e.g., Barry and Wentzel 2006; Piehler and Dishion 2007). It is therefore important that research also focuses on the quality of interactions rather than solely on specific types of relationships (Berndt 2002). In the current study, dyadic mutuality was used to describe the variation in interaction quality between classmates. Given that there are various relations between classmates (e.g., (un)reciprocal friendships, popularity, conflictual relations; Juvonen and Ho 2008), dyadic mutuality is eminently suited to examine the extent to which adolescents are affected by peer influences in the class as it can be used to describe a variety of relations (Piehler and Dishion 2007).

### Classroom Context

As most research examining peer influences on adolescents’ behaviors has mainly focused on the influence of friends (e.g., Dishion et al. 1996; Barry and Wentzel 2006), it is important to broaden the ecological validity of previous findings to the influences of not self-selected peer groups, such as classmates. The classroom peer context is a peer group that is not selected by the adolescent, but with which adolescents have to interact on a daily basis. Studying involuntary, not self-selected peer groups enables examining peer influences beyond selection effects (Juvonen and Ho 2008). This is pivotal since adolescents are exposed to the behaviors of all their classmates, not only to a selective group of classmates (e.g., friends, popular students; Busching and Krabé 2020).

Moreover, the classroom peer context is eminently suited to examine both deviant and prosocial peer influences. Given that the classroom peer context is a not self-selected peer group, there is a wider variety of deviant and prosocial behaviors than in self-selected peer groups (Busching and Krabé 2020). Although previous research examining peer influences often focused on either deviant or prosocial influences (e.g., Hofmann and Müller 2018; Juvonen and Ho 2008), in the present study both types of influences were examined in order to make a direct comparison between the two types of peer influences.

## Current Study

The current study had two aims. The first aim was to study the relation between changes in classmates’ modeling or reinforcement and adolescents’ perceived peer context in the classroom. These *changes* were induced by an intervention, in an experimental field study (e.g., Thomaes et al. 2009). Experimental manipulation strengthens the case that changes in the predictors may be responsible for changes in the outcomes (Kazdin 2007). To this end, the intervention Rock and Water (R&W; Ykema 2002, 2018) was implemented as it aims to decrease deviant and increase prosocial modeling and reinforcement in the class. During the intervention lessons, the mechanisms of modeling and reinforcement are explicitly discussed and prosocial behaviors are reinforced. It was hypothesized that a decrease in deviant and an increase in prosocial modeling and reinforcement of classmates would be related to a more positive perception of the peer context in the classroom. Since the implementation of the intervention was a method of manipulation in the present study, the effectiveness of the intervention is beyond the scope of this paper and is described elsewhere (Mertens et al. 2020). The second aim was to examine whether the relations between classmates’ modeling or reinforcement and perceived classroom peer context was moderated by dyadic mutuality. It was hypothesized that adolescents’ perceptions of the classroom peer context is more strongly influenced by classmates’ modeling and reinforcement in dyads with higher levels of mutuality.

## Method

Data for the present study were collected as part of a larger study examining the effectiveness of R&W which is approved by the Ethical Committee of the Faculty of Social and Behavioral Sciences of Utrecht University (FETC17-015; see for protocol Mertens et al. 2018). The trial is registered in the Dutch Trial Registration number NL6371 (old number NTR6554).

### Procedure

Observation assessments were only conducted in a subsample of the larger project. In 6 schools 14 7th grade classes were randomly selected to participate in the observation task using an online random number generator (R&W condition: 7 classes; control condition: 7 classes). Using the same online generator, students were randomly matched in same-sex dyads within their class. Dyads were composed, as recommended by Huenecke and Waas (2010), of same-sex classmates as young adolescents affiliate mostly with classmates of the same sex.

Observations took place before the start and immediately after the intervention (about 4 months later) for the R&W condition and, parallel, at the same time points for the control condition. Additionally, students completed questionnaires at baseline and, about 4 months later, post intervention. Students gave active informed consent for their participation. Parents gave passive informed consent for participation of their child.

### Participants

In total, 152 students (76 dyads) participated in the observation task at baseline. At post measurement, 130 students (65 dyads) participated again in the same dyad as at baseline. Eleven dyads were missing due to absence of one student (absent on the day of measurement *n* = 6, changed school *n* = 4, refused to participate *n* = 1). Missing data of the final sample (*N* = 130) was missing completely at random (Little’s MCAR test: χ^2^(18) = 18.94, *p* = 0.396).

Students were between 11 and 14 years old (*M* = 12.37, *SD* = 0.56). Of these students, 70 (53.8%) were boys, and 85 (66.9%) had a Western background (i.e., both parents born in Europe, North-America, Oceania, Indonesia, or Japan; Central Bureau for Statistics 2020). The R&W condition consisted of 62 students of whom 34 (54.8%) were boys with an average age of 12.34 (*SD* = 0.63). Of these students, 30 (50.8%) had a Western background. The control condition consisted of 68 students of whom 36 (52.9%) were boys with an average age of 12.40 (*SD* = 0.49). Of these students, 55 (80.9%) had a Western background. An ANOVA and two Chi-squared tests showed no differences between the R&W and control condition concerning age and gender. The conditions differed slightly concerning students’ background (χ^2^(1) = 12.88, *p* < 0.001, φ = 0.318); in the control condition more students had a Western background than in the R&W condition. There were no differences at baseline between the R&W and control condition concerning deviant and prosocial modeling and reinforcement, and outcome variables.

### Conditions

#### Intervention

The theory of the intervention is based on the “R&W house” (Ykema 2002, 2018). This house consists of 5 levels representing modules in which R&W aims to increase students’ experienced safety, to learn students to deal appropriately with difficult situations, to teach about (non)verbal communication, help students to develop their own preferences and choices, and to increase self-insight. This theoretical house is built on the three pillars of self-control, self-reflection, and self-esteem. According to the theory, strengthening students’ skills concerning these pillars enables students to develop themselves within the broader domains of the R&W house (see for more information about the intervention Mertens et al. 2018).

R&W lessons were provided by teachers, mostly physical education teachers, who have followed the 3-day training course to become a certified R&W trainer. The rest of the teaching staff at the school received a 3-day training course to learn how they can support the R&W trainers and how to implement R&W during their regular classes.

Students received 14 weekly lessons of 90 min during a four months period. The lessons were provided during physical education due to the physical nature of R&W as it combines a physical approach with a psychological approach, i.e., a psychophysical approach. That is, students learn through play and exercises how to make (physical) contact with others, and explore, respect, and set own and other’s boundaries. Each lesson is described in the manual and includes physical exercises, reflection, a moment of sharing thoughts with each other, and an exercise to strengthen the transfer of the learned skills to students’ daily life.

#### Control

Students from the three schools in the control condition received care as usual. In one school this entailed a mentor students can go to with problems, a project week about ‘being different’ and an anti-bullying protocol. Another school had an anti-bullying coordinator and assigned a personal coach to each student with whom the student had regular meetings for advice and discussing the student’s wellbeing. The third school also had an anti-bullying coordinator and a mentor students could to go to.

### Measurements

#### Perceived classroom peer context

Levels of comfort, cohesion, and conflict in the class were measured with three subscales of the Classroom Peer Context questionnaire (Boor-Klip et al. 2016). The subscale Comfort assesses the level to which students feel at ease around their classmates (e.g., “In this class I can be myself.”), Cohesion assesses unity and inclusiveness among classmates (e.g., “In this class children like each other.”), and Conflict assesses students’ negative social exchanges in the classroom (e.g., “In this class children fight with each other.”). Each subscale contained 4 items answered on a 5-point Likert-type scale (1 = *totally not true*, 5 = *completely true*). Cronbach’s α was for Comfort 0.71 and 0.84 for T1 and T2, respectively, for Conflict 0.83 and 0.88, and for Cohesion 0.44 and 0.62. Due to the relatively low reliability of Cohesion, this construct was not analyzed.

Experienced victimization was assessed with 1 item of the global measures of the Olweus Bully/Victim Questionnaire (Solberg and Olweus 2003): “How often have you been bullied at school in the past two months?”. This item was preceded by a definition of bullying. Frequency was indicated on a 5-point scale (1 = *never*, 5 = *multiple times a week*).

#### Deviant and prosocial modeling and reinforcement

Observations of peer interactions took place at the students’ school in a private room during school hours and were videotaped by trained research assistants. The research assistant explained the procedure of the observation task, guaranteed confidentiality, and pointed out the option to withdraw from the task. The research assistant was not present in the observation room during the discussions to enable the dyad to talk freely and kept track of time outside the observation room. To minimize unintended influences, students were asked at the end of the observation not to tell their classmates about the task until all students had participated.

The observation task was based on the Peer Interaction Task (e.g., Dishion et al. 1996). The interaction consisted of four vignettes which students each discussed for 5 min. The first vignette was planning an activity together as a warm-up. The other three vignettes, systematically counterbalanced, concerned daily school situations involving: Student at work in the class, student with new clothes, and sitting together with classmates. For example, “classmate A is in the classroom working on an assignment in his book. Classmate B is doing nothing. Classmate B is annoying and throws pieces of paper towards classmate A.” Two different versions of all vignettes were used for the baseline and post measurements. Each participant received the vignettes on paper. They were instructed to read the vignettes in turn aloud and discuss the situation together for 5 min. After the instruction, the research assistant told the students they could begin and left the room. Five minutes later, the research assistant re-entered the observation room to end the discussion and provided the next vignette. In addition to the vignettes, students were given a paper with three questions they could use for each vignette in order to help them discuss the situation for the full 5 min: (1) What do you think of the situation? Could this happen at your school? (2) Imagine you are classmate A. What would you do? (3) How could this end? (The full protocol for the observation task is available from the first author.)

The Conversation topic code (Piehler and Dishion 2004b) was used to assess frequencies of deviant and prosocial verbal and nonverbal modeling. Verbal modeling was coded based on verbatim transcription of the discussion. Deviant modeling was all utterances that violated community or societal rules or were not appropriate to the setting or task (e.g., “I would hit him in his face.”). Prosocial modeling was all utterances referring to positive or prosocial values, principles, or actions (e.g., “I wouldn’t bully him.”). Neutral modeling was all utterances that did not fit in the deviant or prosocial categories (e.g., “This situation happens all the time.”).

Nonverbal modeling was coded, while watching the videotaped observation, when participants used gestures to support their utterance or only used gestures. Depending on the content of the gesture it was coded as deviant (e.g., making a punch movement, making weird faces) or prosocial (e.g., waving their hand as a greeting).

Proportions of (verbal and nonverbal) deviant and prosocial modeling were calculated over all verbal (i.e., prosocial, deviant, and neutral utterances) and nonverbal behaviors during the interaction, representing the proportion of prosocial and deviant modeling relative to all coded modeling. Interrater reliability of the three independent coders was good concerning deviant and prosocial modeling (ICC_deviant_ = 0.96, ICC_prosocial_ = 0.96) based on 22 observations coded over time.

In addition to deviant and prosocial modeling, verbal (e.g., “Indeed”, “True”, “No”) and nonverbal (e.g., laughing, giving thumbs up, shaking head) reactions were coded (Piehler and Dision 2007; Van de Bongardt et al. 2017). Reactions were coded as reinforcement or as correction. Reactions were coded as reinforcement when the reaction indicated a positive evaluation of the other peer’s behavior (e.g., “Indeed”, laughing). Reactions were coded as deviant reinforcement when deviant behavior of the peer was reinforced and as prosocial reinforcement when prosocial behavior of the peer was reinforced. Corrections were coded when the reaction indicated a negative evaluation of the other peer’s behavior (e.g., “No”, shaking head).

Proportions of deviant and prosocial reinforcement were calculated relative to all coded reaction codes (i.e., reinforcements and corrections). Interrater reliability of the three independent coders was good concerning deviant and prosocial reinforcement (ICC_deviant_ = 0.82, ICC_prosocial_ = 0.87) based on 22 observations coded over time.

#### Dyadic Mutuality

Dyadic mutuality was assessed at T1, through the video-observations, based on the *peer dyadic mutuality rating system* (Piehler and Dishion 2004a). Each member of the dyad was coded on three items: Responsiveness (i.e., the extent to which the student responded verbally and nonverbally to his or her peer), self-centeredness (i.e., the extent to which the student redirected the conversational flow to focus on personal ideas and experiences), and communicative efficiency (i.e., the appropriateness and competence of the messages send during the discussion—item added to the rating system; Whalen et al. 1979).

Additionally, each dyad as a whole was coded on three items: Reciprocity (i.e., verbal reciprocity such as engaging in a conversation-like interaction, and behavioral reciprocity such as eye-contact and posture orientation), shared attitudes and values (i.e., similar beliefs and attitudes about the discussed ideas) and affective valence (i.e., the emotional tone of the discussion and nonverbal behavior such as gestures, facial expression, and tone of voice.

All items were rated on a 6-point Likert type scale (1 = *rarely or never*, 6 = *always or throughout*) and coded for the session as a whole. The item self-centeredness was reversed coded, so high values representing low self-centeredness. Subsequently, the 9 items (i.e., two times three individual items and three items of the dyad) were averaged to form a score on dyadic mutuality per dyad. Interrater reliability of the three independent coders was good (ICC = 0.73) based on 22 observations coded over time.

### Analyses

**Fig. 1:**
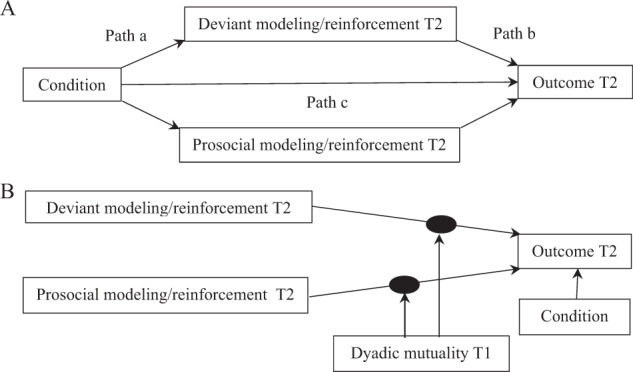
**a** Multilevel model of changes in deviant and prosocial modeling/reinforcement as predictor of the outcome (i.e., comfort, conflict, and victimization) at classroom level with an intervention as manipulation. The examined relations were not significant, except for the relation between prosocial modeling and experienced victimization (*B* = −1.86; 95%CI [−3.23; −0.49]). **b** Multilevel model with dyadic mutuality as moderator of the relation between deviant and prosocial modeling/reinforcement and the outcome (i.e., comfort, conflict, and victimization). Only the relation between prosocial modeling and experienced victimization was moderated indicating a stronger relation with higher levels of dyadic mutuality

First, it was tested whether changes in students’ modeling or reinforcement were related to adolescents’ perceived classroom peer context in M*plus* 8.2. Multilevel models were modeled in order to analyze changes in modeling, reinforcement, and perceived classroom peer context at classroom level (Preacher et al. 2010). The intervention (i.e., condition) was an independent variable that served as a predictor of change in modeling or reinforcement and the outcome. Deviant and prosocial modeling were analyzed in parallel in one model per outcome measure. Likewise, deviant and prosocial reinforcement were analyzed in parallel in one model per outcome measure (see Fig. 1a). Baseline measures of the concerned variables were added as covariates. Additionally, since the conditions differed significantly on ethnic background, this variable was added as a covariate. If ethnic background was a significant covariate it was retained in the model, otherwise it was dropped in favor of a more parsimonious model.

Second, it was tested whether the relation between changes in modeling and reinforcement and adolescents’ perceived classroom peer context was moderated by dyadic mutuality, using multilevel analyses in M*plus* 8.2. At level 1, the individual level, within-dyad variation between modeling or reinforcement and the outcome was modeled. This relation was allowed to vary between individuals using a random slope. At level 2, the dyad level, dyadic mutuality was examined as predictor of the variation in the mean slope of modeling or reinforcement and the outcome. Deviant and prosocial modeling were analyzed in parallel in one model per outcome, as were deviant and prosocial reinforcement. Condition was added at level 2 as a predictor of the outcome to control for change in adolescents’ perceived classroom peer context explained by the condition in which the adolescents participated. Baseline measures of modeling/reinforcement and the concerned outcome were added as covariates (see Fig. 1b). Due to the estimation of cross-level interactions, no standardized fit indices were available. There is significant moderation when the slope between modeling or reinforcement and the outcome is dependent on the level of dyadic mutuality. In case of a significant moderation, the differing relations for dyads with low, average, and high levels (*M* ± 1 *SD*) of dyadic mutuality between the concerning independent variable and outcome were graphically displayed.

## Results

### Preliminary Analyses

Group differences post intervention on the perceived classroom peer context, modeling, and reinforcement were examined using ANCOVAs, controlling for ethnicity and the corresponding baseline measure (see Table 1). No significant differences between the conditions were found. Correlations between the variables are reported in the Appendix.

**Table 1:** Descriptives of students’ perceived classroom peer context, modeling, reinforcement, and dyadic mutuality per condition and group comparison at post-test

	R&W		Control		Post-test differences (ANCOVA)		
	Baseline *M* (*SD*)	Post-test *M* (*SD*)	Baseline *M* (*SD*)	Post-test *M* (*SD*)	*F*	*p*	η^2^_partial_
Outcomes							
Comfort	4.35 (0.80)	4.19 (1.04)	4.57 (0.56)	4.36 (0.76)	0.23	0.633	0.002
Cohesion	4.27 (0.72)	4.05 (0.95)	4.36 (0.65)	4.23 (0.69)	0.04	0.850	0.000
Conflict	2.58 (1.15)	2.43 (1.24)	2.31 (1.00)	2.39 (1.02)	0.44	0.508	0.004
Victimization	1.29 (0.67)	1.32 (0.86)	1.18 (0.60)	1.14 (0.43)	1.32	0.253	0.011
Mediators							
Deviant modeling	0.27 (0.17)	0.34 (0.21)	0.25 (0.14)	0.33 (0.15)	0.11	0.742	0.001
Prosocial modeling	0.17 (0.11)	0.15 (0.11)	0.16 (0.09)	0.14 (0.08)	0.67	0.417	0.005
Deviant reinforcement	0.30 (0.28)	0.38 (0.33)	0.29 (0.21)	0.43 (0.27)	0.91	0.343	0.007
Prosocial reinforcement	0.46 (0.34)	0.39 (0.34)	0.45 (0.28)	0.33 (0.28)	1.79	0.183	0.014
Moderator							
Dyadic mutuality	4.27 (0.57)		4.51 (0.53)				

### Classmates’ Modeling and Reinforcement

The intervention did not predict changes in modeling or reinforcement (Path a; see Table 2). Changes in modeling or reinforcement also did not predict the outcomes, except victimization (Path b). An increase in prosocial modeling was related to a decrease in experienced victimization.

**Table 2:** Effects of modeling and reinforcement as predictors at classroom level

	Modeling						Reinforcement					
	Path a		Path b		Indirect effect		Path a		Path b		Indirect effect	
	B (SE)	95% CI	B (SE)	95% CI	Effect (SE)	95% CI	B (SE)	95% CI	B (SE)	95% CI	Effect (SE)	95% CI
Model 1 Comfort												
Deviant	−0.00 (0.03)	−0.06; 0.06	0.06 (0.36)	−0.64; 0.76	0.00 (0.00)	−0.00; 0.00	−0.06 (0.07)	−0.19; 0.07	−0.26 (0.43)	−1.11; 0.59	0.02 (0.03)	−0.04; 0.07
Prosocial	0.02 (0.01)	−0.00; 0.04	0.41 (0.92)	−1.40; 2.21	0.01 (0.02)	−0.02; 0.04	0.07 (0.07)	−0.07; 0.21	−0.10 (0.31)	−0.71; 0.52	−0.01 (0.02)	−0.05; 0.04
Model 2 Cohesion												
Deviant	−0.00 (0.03)	−0.06; 0.06	−0.31 (0.41)	−1.11; 0.48	0.00 (0.01)	−0.02; 0.02	−0.06 (0.06)	−0.17; 0.05	−0.21 (0.34)	−0.88; 0.45	0.01 (0.02)	−0.03; 0.06
Prosocial	0.01 (0.01)	−0.01; 0.03	0.39 (0.76)	−1.10; 1.88	0.01 (0.01)	−0.02; 0.03	0.06 (0.05)	−0.04; 0.16	0.00 (0.36)	−0.71; 0.72	0.00 (0.02)	−0.04; 0.04
Model 3 Conflict												
Deviant	0.00 (0.03)	−0.06; 0.06	−0.02 (0.50)	−0.99; 0.95	0.00 (0.00)	−0.00; 0.00	−0.07 (0.06)	−0.17; 0.04	0.33 (0.44)	−0.53; 1.18	−0.02 (0.03)	−0.08; 0.04
Prosocial	0.02 (0.01)	−0.01; 0.04	−0.51 (0.91)	−2.30; 1.28	−0.01 (0.01)	−0.04; 0.02	0.06 (0.05)	−0.03; 0.16	0.12 (0.29)	−0.45; 0.69	0.01 (0.02)	−0.03; 0.04
Model 4 Victimization												
Deviant	−0.00 (0.03)	−0.06; 0.06	−0.15 (0.48)	−1.09; 0.80	0.00 (0.00)	−0.01; 0.01	−0.05 (0.08)	−0.20; 0.11	0.21 (0.46)	−0.69; 10.10	−0.01 (0.02)	−0.05; 0.03
Prosocial	0.01 (0.01)	−0.01; 0.04	−1.86 (0.70)^**^	−3.23; −0.49	−0.03 (0.02)	−0.07; 0.02	0.06 (0.36)	−0.90; 7.75	−0.27 (0.25)	−0.76; 0.21	−0.02 (0.10)	−0.22; 0.19

Path a: Condition → Modeling/Reinforcement; Path b: Modeling/reinforcement → Outcome; **p < 0.01

### Dyadic Mutuality

**Fig. 2:**
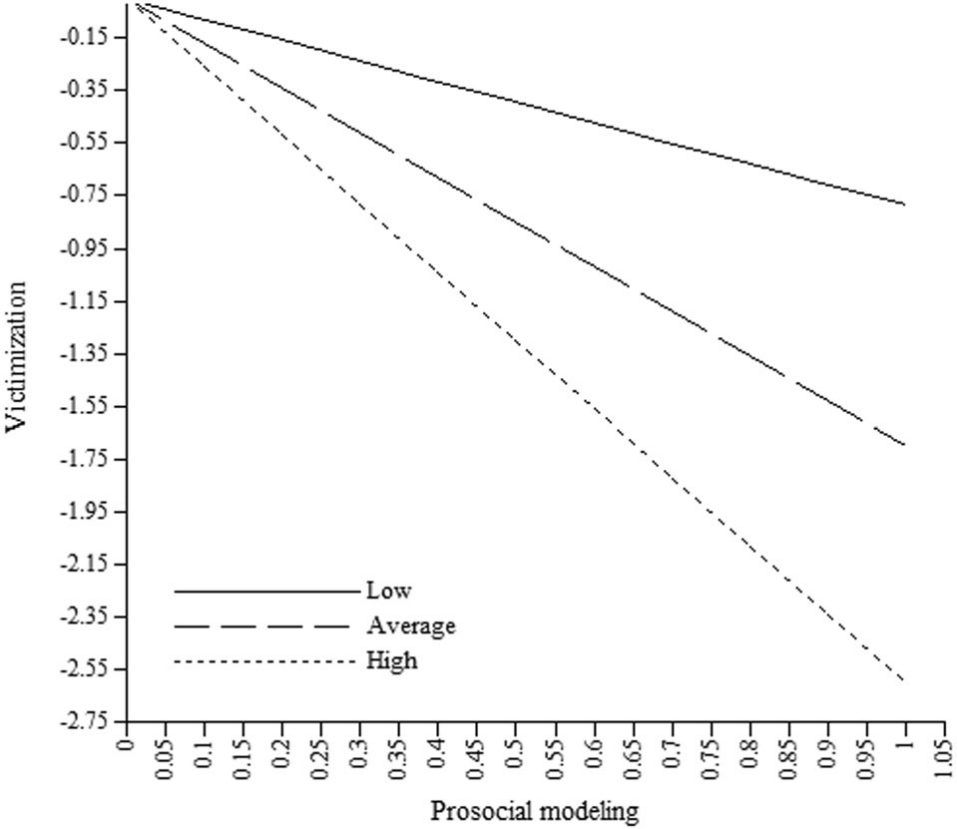
Moderation of dyadic mutuality of the relation between prosocial modeling and victimization

Dyadic mutuality moderated the relation between changes in prosocial modeling and experienced victimization (see Table 3). The negative relation between changes in prosocial modeling and experienced victimization was stronger for dyads with higher levels of mutuality (*B*_*Low*_ = −0.79; *B*_*Average*_ = −1.70; *B*_*High*_ = −2.60; see Fig. 2). No other moderations by dyadic mutuality were found on either modeling or reinforcement.

**Table 3:** Moderation of dyadic mutuality of the relation between modeling or reinforcement and perceived classroom peer context at the dyad level

	Modeling		Reinforcement	
	*B* (*SE*)	95% CI	*B* (*SE*)	95% CI
Model 1 Comfort				
Deviant × mutuality	1.28 (2.48)	−3.58; 6.14	1.19 (0.61)	−0.01; 2.39
Prosocial × mutuality	2.80 (3.71)	−4.48; 10.07	−0.31 (0.57)	−1.43; 0.81
Model 2 Cohesion				
Deviant × mutuality	1.26 (1.15)	−0.99; 3.50	0.84 (0.53)	−0.19; 1.87
Prosocial × mutuality	3.22 (2.04)	−0.79; 7.22	−0.25 (0.53)	−1.28; 0.79
Model 3 Conflict				
Deviant × mutuality	−2.43 (1.67)	−5.69; 0.84	−0.03 (0.61)	−1.23; 1.16
Prosocial × mutuality	−2.37 (1.55)	−5.40; 0.66	−0.01 (0.56)	−1.10; 1.08
Model 4 Victimization				
Deviant × mutuality	0.06 (0.54)	−0.99; 1.12	0.24 (0.30)	−0.35; 0.84
Prosocial × mutuality	−1.61 (0.57)^**^	−2.73; −0.48	−0.02 (0.20)	−0.41; 0.37

*p < 0.05; **p < 0.01

## Discussion

Most research on peer influences has focused on peer influences of self-selected peer groups, i.e., friends. Less is known about peer influences of involuntary selected peer groups, i.e., classmates. It is important to study classmates’ influences since experiences with peers in the classroom can affect adolescents’ academic, emotional, and social development (Rubin et al. 2006). The present study examined deviant as well as prosocial influences of classmates within an experimental design (i.e., manipulation by a school-based intervention). The results suggest that classmates’ prosocial influences may be more influential concerning adolescents’ perceived classroom peer context than classmates’ deviant influences. No relations between changes in classmates’ deviant modeling or reinforcement and changes in the perceived peer context in the class were found. Increases in classmates’ prosocial modeling, though, were related to decreases in experienced victimization, especially when levels of dyadic mutuality between classmates were high. This means that it might be worthwhile for interventions aiming to decrease victimization to stimulate prosocial modeling in the class, although it should be noticed that changes in classmates’ prosocial modeling in the present study were not induced by the intervention, as the degree of change was similar in the intervention and the control condition.

The finding that an increase in prosocial modeling is related to a decrease in victimization is in line with the social learning theory (Bandura 1978) and has important implications for anti-bullying interventions. Classmates showing prosocial modeling are more inclined to show affiliation and involve all classmates in classroom activities degrading the level of exclusion and rejection (Juvonen and Ho 2008). Other students might imitate this prosocial behavior which results in less victimization. In contrast to Ando and colleagues (2005), no relation was found between deviant peer influences and victimization. Given that Ando and colleagues (2005) studied peer influences of friends and the present study influences of classmates, the difference in findings might indicate that the processes through which friends influence each other differ from the processes through which classmates influence each other. Thus, for diminishing victimization in the classroom specifically, it seems important to focus on stimulating prosocial behaviors rather than reducing deviant behaviors, which is in line with suggestions of Busching and Krahé (2020).

The relation between prosocial modeling and victimization appears to be stronger when dyadic mutuality levels between classmates are higher. In interactions in which classmates are interested in each other, show affection, and are responsive, students appear to be more influenced by the prosocial behaviors of their classmates and might be more likely to imitate these behaviors, which is in line with previous research (e.g., Barry and Wentzel 2006). Hence, in addition to stimulating prosocial behaviors in the class, attention should be given to improving classmates’ mutuality in order to strengthen the positive effect of prosocial modeling on victimization. For instance, interventions could provide positive and fun exercises in the class in which classmates who do not interact on a daily basis work together. This might improve students’ emotions toward each other and their expectations for future interactions (Rubin et al. 2006) resulting in more positive mutual feelings and affection between students.

No relations between deviant and prosocial modeling or reinforcement and interpersonal relations in the class (i.e., perceived levels of comfort and conflict) were found. Perhaps, victimization might more strongly represent adolescents’ perceptions of the school context as dangerous (Goldstein et al. 2008), whereas interpersonal relations might more strongly represent adolescents’ feelings of social support in the classroom (Hopson et al. 2014). While perceptions of the school context appear to be influenced by experiencing and witnessing the behaviors of all classmates (Goldstein et al. 2008), perceived social support appears to be mostly influenced by friends (Bokhorst et al. 2010). Hence, maybe only modeling and reinforcement of friends in the class have an influence on perceived interpersonal relations in the class.

The absence of a relation between modeling or reinforcement and interpersonal relations in the class could also have a methodological explanation. The questionnaire regarding interpersonal relations in the class consisted of items referring to the class and classmates in general (i.e., assessing adolescents’ perceptions of their classmates in general), whereas the question regarding experienced victimization concerned the adolescents themselves. For instance, when adolescents indicated that there were conflicts in the class, they were not necessarily involved in these conflicts. In contrast, when adolescents indicated experienced victimization by bullies they were victimized themselves. Even though the used questionnaire gives a general overview of the interpersonal relations in the class, it does not indicate to what extent adolescents themselves are affected by the interactions and relations between classmates. Thus, future research examining interpersonal relations in the class should add questions asking to which extent adolescents are affected by the behaviors of other classmates.

The finding that the intervention did not change classmates’ modeling and reinforcement might indicate that modeling and reinforcement were not intensely enough addressed during this intervention or that the intervention’s time span was too short. More intensive attention to setting negative consequences for deviant behaviors and reinforcing prosocial behaviors in the classroom has been related to more positive behaviors of students (Phillips Smith et al. 2006). This classroom management approach is used during the intervention lessons, but may not have been used during regular lessons, limiting the opportunities for students to learn from prosocial models and refrain from deviant models (Phillips Smith et al. 2006). Another explanation might be that the intervention’s time span of approximately four months is too short. Studies showing a relation between peer influence and antisocial behavior use a time span from six months to one year (Sijtsema and Lindenberg 2018). Hence, an intervention time span of at least six months may be necessary to establish change in classmates’ modeling and reinforcement.

When considering the findings of the current study, it is important to note some strengths and limitations. A strength of the study is the use of observations to assess classmates’ influences and mutuality. Using observations modeling, reinforcement, and dyadic mutuality were directly coded without depending on subjective perspectives of students. Furthermore, both deviant and prosocial peer influences were examined in not self-selected dyads. This allowed us to examine negative as well as positive peer influences with reduced selection effects. Moreover, the present study had an experimental design with a pre- and post-measurement enabling us to examine *changes* in modeling, reinforcement, and the perceived classroom peer context.

A limitation of the present study is the somewhat small sample size. Even though the sample size is rather large for an observation study, it might be that due to the relatively small sample size some relations failed to reach significance. Additionally, no classroom characteristics were examined as predictors. Due to the limited number of clusters at classroom level the models were kept as simple as possible. However, classmates’ influences might depend on characteristics of the general classroom context such as class size or gender composition. Future research could focus on classroom characteristics and examine whether these characteristics influence adolescents’ perceived classroom peer context. Moreover, modeling, reinforcement, and the classroom peer context were measured at the same time point (i.e., post intervention). This time point was analyzed because the interest was in changes in modeling and reinforcement which were expected immediately after the intervention rather than between post and follow-up measurements (Beauchaine and Slep 2018). However, this approach limited the extent to infer causal order (Weeland et al. 2018). Furthermore, victimization was measured with only one item. Even though it is common in research concerning bullying to measure (types of) victimization with one item, it might be more reliable to use multiple items. In addition, the reliability of cohesion in the class was relatively low due to which this construct was not analyzed. Future research should aim to reliably measure cohesion and examine classmates’ influences on cohesion in the class.

## Conclusion

For (early) adolescents, the class is an important social context and classmates can affect adolescents’ academic, emotional, and social development (Rubin et al. 2006). Yet much research on peer influences has focused on influences of friends rather than of classmates. The current study examined classmates’ deviant and prosocial influences on adolescents’ perceived peer context in the class in a field experiment with observations. Although no relations between classmates’ modeling or reinforcement and interpersonal relations in the class (i.e., comfort and conflict between classmates) were found, the results did show that an increase in classmates’ prosocial modeling was related to a decrease in victimization in the class, especially when dyadic mutuality was high. These results suggest that classmates’ prosocial behaviors rather than their deviant behaviors can affect adolescents’ perceptions. This finding underscores the importance of examining both prosocial and deviant behaviors within the same study as they are not mere ends of the same continuum. Extending knowledge of peer influences of classmates on early adolescents (12–13 years) is important. While among friends deviant behaviors seem important peer influences, among classmates especially prosocial behaviors seem to have impact.

## Appendix

**Table 4:** Correlations between variables assessed at baseline and post intervention

	1.	2.	3.	4.	5.	6.	7.	8.	9.	10.	11.	12.	13.	14.	15.	16.	
Classroom peer context T1																	
1. Comfort	–																
2. Cohesion	0.45**	–															
3. Conflict	−0.23**	−0.51**	–														
4. Victimization	0.15	−0.42**	−0.15	–													
Mediators T1																	
5. Deviant modeling	0.26**	−0.15	−0.27**	−0.03	–												
6. Prosocial modeling	−0.21*	0.13	0.22*	−0.14	−0.46**	–											
7. Deviant reinforcement	0.13	−0.06	−0.08	−0.01	0.36**	−0.26**	–										
8. Prosocial reinforcement	−0.23**	0.03	0.17	0.02	−0.49**	0.36**	−0.64**	–									
Moderators T1																	
9. Dyadic mutuality	−0.22*	0.00	0.07	−0.12	0.04	0.02	0.05	0.02	–								
Classroom peer context T2																	
10. Comfort	−0.37**	0.30**	0.33**	−0.10	−0.15	0.20*	−0.15	0.14	−0.16	−0.25**	–						
11. Cohesion	−0.38**	0.27**	0.37**	−0.09	−0.14	0.28**	−0.18	0.10	0.15	−0.36**	73**	–					
12. Conflict	−0.23**	−0.07	0.30**	0.11	−0.02	−0.05	−0.17	0.09	0.14	−0.25**	−0.36**						
13. Victimization	0.01	−0.15	−0.11	0.09	0.19*	−0.18*	−0.02	−0.23**	0.08	0.25**	−0.12	−0.07	–				
Mediators T2																	
14. Deviant modeling	0.27**	−0.03	−0.14	0.03	0.48**	−0.40**	0.15	−0.25**	−0.24**	0.14	−0.11	−0.18	0.14	–			
15. Prosocial modeling	−0.12	0.19*	0.16	−0.14	−0.36**	0.51**	−0.21*	0.26**	0.03	−0.09	0.19*	0.18	−0.26**	−0.54**	–		
16. Deviant reinforcement	0.10	0.08	−0.05	−0.08	0.13	−0.15	0.06	−0.15	−0.07	0.14	−0.05	−0.11	0.13	0.39**	−0.20*	–	
17. Prosocial reinforcement	−0.15	0.02	0.08	0.03	−0.27**	0.20*	−0.14	0.28**	0.10	−0.15	0.11	0.13	−0.22*	−0.43**	0.18*	−0.61**	

**p* < 0.05; ***p* < 0.01
